# *De novo* Assembly and Characterization of the Barnyardgrass (*Echinochloa crus-galli*) Transcriptome Using Next-Generation Pyrosequencing

**DOI:** 10.1371/journal.pone.0069168

**Published:** 2013-07-10

**Authors:** Xia Yang, Xin-Yan Yu, Yong-Feng Li

**Affiliations:** Institute of Plant Protection, Jiangsu Academy of Agricultural Sciences, Nanjing, China; University of Toronto, Canada

## Abstract

**Background:**

Barnyardgrass (*Echinochloa crus-galli*) is an important weed that is a menace to rice cultivation and production. Rapid evolution of herbicide resistance in this weed makes it one of the most difficult to manage using herbicides. Since genome-wide sequence data for barnyardgrass is limited, we sequenced the transcriptomes of susceptible and resistant barnyardgrass biotypes using the 454 GS-FLX platform.

**Results:**

454 pyrosequencing generated 371,281 raw reads with an average length of 341.8 bp, which made a total length of 126.89 Mb (SRX160526). *De novo* assembly produced 10,142 contigs (∼5.92 Mb) with an average length of 583 bp and 68,940 singletons (∼22.13 Mb) with an average length of 321 bp. About 244,653 GO term assignments to the biological process, cellular component and molecular function categories were obtained. A total of 6,092 contigs and singletons with 2,515 enzyme commission numbers were assigned to 151 predicted KEGG metabolic pathways. Digital abundance analysis using Illumina sequencing identified 78,124 transcripts among susceptible, resistant, herbicide-treated susceptible and herbicide-treated resistant barnyardgrass biotypes. From these analyses, eight herbicide target-site gene groups and four non-target-site gene groups were identified in the resistant biotype. These could be potential candidate genes involved in the herbicide resistance of barnyardgrass and could be used for further functional genomics research. C_4_ photosynthesis genes including *RbcS*, *RbcL*, *NADP-me* and *MDH* with complete CDS were identified using PCR and RACE technology.

**Conclusions:**

This is the first large-scale transcriptome sequencing of *E. crus-galli* performed using the 454 GS-FLX platform. Potential candidate genes involved in the evolution of herbicide resistance were identified from the assembled sequences. This transcriptome data may serve as a reference for further gene expression and functional genomics studies, and will facilitate the study of herbicide resistance at the molecular level in this species as well as other weeds.

## Introduction

Barnyardgrass (*Echinochloa crus-galli* [L.] Beauv.) is one of the main problematic grass weeds that grows along with important staple crops such as rice [1]. During cultivation even when the ratio of rice plants to barnyardgrass is 10∶1 rice biomass is reduced by 75% and yield by about 50% [2]. Many herbicides are being used to destroy barnyardgrass which in turn would improve rice production. However, persistent use of herbicides results in rapid development of herbicide resistance [3]–[4]. In the last two decades, it has been reported that *E. crus-galli* worldwide has developed resistance to nine herbicide groups: ALS inhibitors (e.g. penoxsulam, bispyribac-sodium), ACCase inhibitors (e.g. cyhalofop-butyl), synthetic auxin (e.g. quinclorac), photosystem II (e.g. atrazine), ureas and amides (inhibition of photosynthesis at photosystem II, e.g. propanil), dinitroanilines (microtubule assembly inhibition, e.g. pendimethalin), thiocarbamates (inhibition of lipid synthesis, e.g. thiobencarb), chloroacetamides (inhibition of cell division, e.g. butachlor) and isoxazolidinoes (inhibition of carotenoid biosynthesis, e.g. clomazone) [4]–[8]. The increasing resistance of *E. crus-galli* to herbicides has drastically threatened rice production and alternate weed management strategies should be considered during the cultivation of direct-seeded rice. Therefore, an understanding of the fundamental molecular mechanisms behind development of herbicide resistance is necessary to minimize and manage resistance development, and increase crop yield [3]–[4].

Recent advances in genomic sequencing technologies are radically changing biological research, and will also have a major impact on crop improvement [9]–[11]. However, genomics and bioinformatics studies on the rapidly evolving weeds of modern agriculture are limited [12]. Therefore, developing new genomics resources is necessary to study the troublesome weeds in crop fields.

In recent years, the next generation sequencing (NGS) technologies have enabled inexpensive and quick generation of large-scale sequence data when compared to conventional Sanger sequencing [9], [13]. The 454 pyrosequencing technology has been used to analyze the transcriptome of grass species such as *Brachypodium distachyon*, waterhemp (*A. tuberculatus)*, horseweed (*C. canadenisis)*, grain amaranth (*A. hypochondriacus*), 11 composite weeds and switchgrass (*Panicum virgatum*) [14]–[19]. Besides obtaining the transcriptomic data from these species, some candidate genes potentially involved in herbicide resistance have also been analyzed. The preliminary sequence data for all of the major target-site genes such as 4-hydroxyphenylpyruvate dioxygenase and glutamine synthetase were obtained in *A. tuberculatus* [15]. The functions of ABC transporters involved in non-target glyphosate resistance in *C. canadenisis* were demonstrated by real-time PCR experiments [16]. These data indicate that 454 GS-FLX pyrosequencing is a powerful tool for the development of genomic resources for functional genomics on grasses. They would also potentially benefit future research efforts in weed science.

To increase the genomic resources for weeds, we sequenced the trancriptomes of the susceptible and resistant biotypes of barnyardgrass (*E. crus-galli*) using the 454 GS-FLX platform. The data were assembled *de novo* followed by sequence annotation and clustering into putative functional categories using the Gene Ontology (GO) framework and grouping into pathways using the Kyoto Encyclopedia of Genes and Genomes (KEGG) database. Moreover, transcript abundance analysis was performed using Illumina sequencing and the observed differences between the resistant and susceptible *E. crus-galli* biotypes are presented here. Then, potential candidate sequences involved in the development of herbicide resistance were also analyzed. Finally, C_4_ photosynthesis genes including *RbcS*, *RbcL*, *NADP-me* and *MDH* with complete CDS were identified using PCR and RACE technology.

*De novo* sequence assembly was then performed, which involved comparison of sequences, finding overlapping fragment pairs and merging as many fragments as possible to create a consensus sequence [27]. This assembly resulted in 10,142 contigs (∼5.92 Mb) with an average length of 583 bp and 68,940 singletons (∼22.13 Mb) with an average length of 321 bp (Table 1). The length distribution of all assembled contigs and singletons is shown in Fig. 3. About 9,432 contigs and 64,272 singletons were >100 bp, 5,103 (50.32%) contigs were longer than 500 bp and 2,935 (28.94%) were longer than 700 bp. The longest contig and singleton was 6,708 and 878 bp respectively. The N50 value for the contigs was 900 bp and represents the contig length of half of the total contigs.

**Table 1:** Summary of barnyardgrass 454 transcriptome sequencing, assembly and annotation.

	Reads (n)	Bases (Mb)	Average length (bp)
**454 raw reads**	371,281	126.89	341
**Assembled contigs**	10,142	5.92	583
**Singletons**	68,940	22.13	321
**Annotated sequences**	45,438	18.92	416
**Sequences assigned with GO terms**	40,399	17.03	421
**Sequences assigned with EC numbers**	12,846	5.72	445

## Materials and Methods

We promise that no specific permissions were required for the barnyardgrass species in the described locations in this manuscript, and the field studies did not involve endangered or protected species.

### Plant Materials and Growth

**Figure 1:**
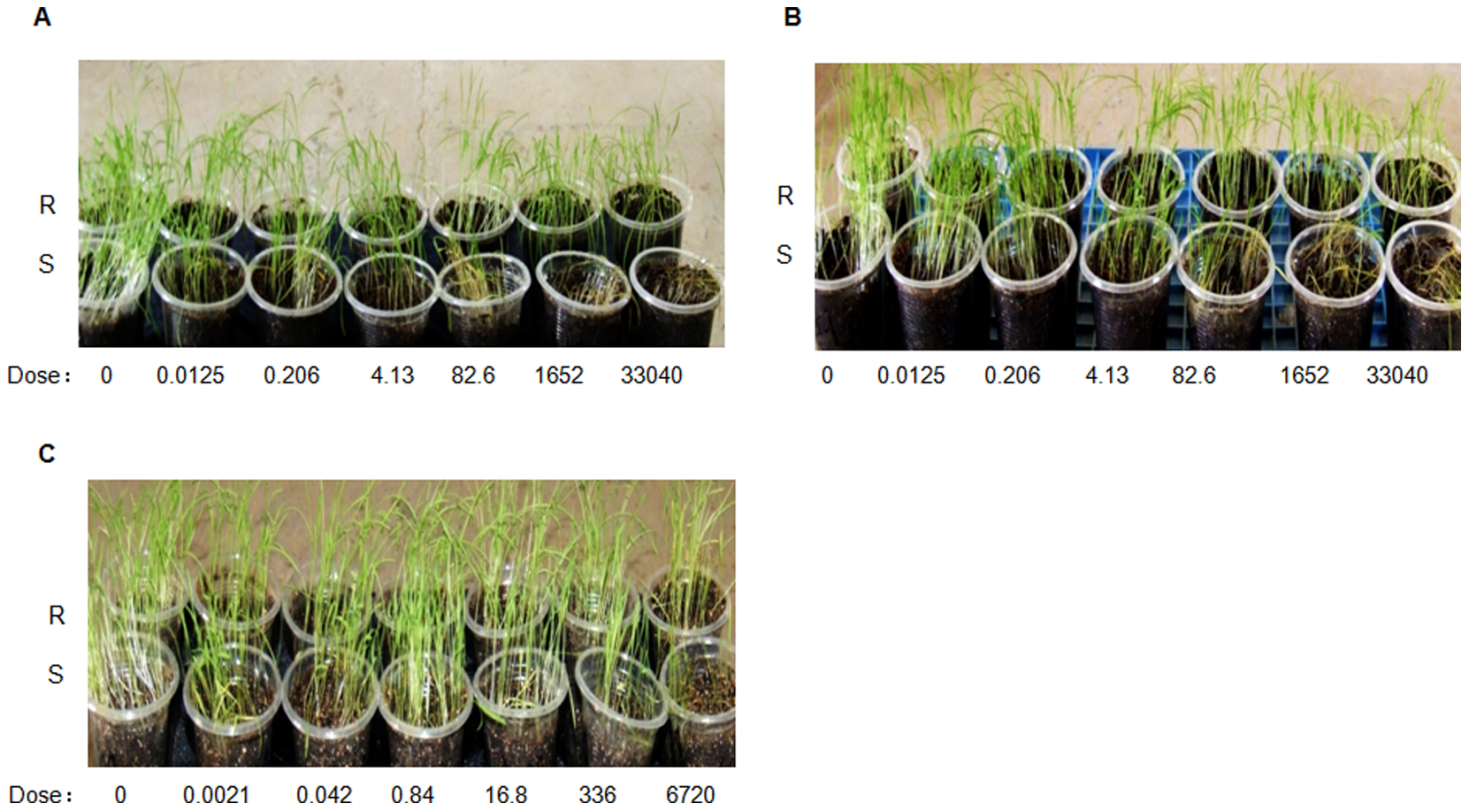
The growth of resistant (R) and susceptible (S) barnyardgrass biotypes at various concentrations of the herbicides quinclorac (A), bispyribac-sodium (B) and penoxsulam (C). The dose of each herbicide is in µmol.

Barnyardgrass (*E. crus-galli*) seeds from susceptible and resistant biotypes were collected from the rice field at Lu-jiang county in An-hui province, China. Before the experiments, this resistant biotype was first confirmed to be resistant to quinclorac (inhibitor of synthetic auxin and also inhibitor of cell wall), penoxsulam (ALS inhibitor) and bispyribac-sodium (ALS inhibitor) group of herbicides (Fig. 1). For this, the seeds were sterilized in 3% NaClO for 10 min, washed three times with tap water and allowed to germinate at 30°C. These germinating seeds were then planted in plastic pots, filled with a 2∶1:1 mixture of soil: peat: sand in a climate chamber at 28°C/25°C (day/night) with a 16 h photoperiod. Three weeks later, leaves from the susceptible and resistant biotype seedlings were treated with 1.143 mg/ml quinclorac (BASF AG, German), 4.114 mg/ml penoxsulam (Dow AgroSciences, USA) and 3.429 mg/ml bispyribac-sodium (Jiangsu Institute of Ecomones Co., LTD, China). After 24 h of spraying, both treated and untreated seedlings were harvested and immediately frozen in liquid nitrogen, and stored at −80°C until RNA extraction.

### RNA Isolation and cDNA Library Construction

Total RNA from the seedlings was extracted using Trizol reagent (Invitrogen, USA) according to the manufacturer’s protocol. RNA quantity and quality were analyzed using gel electrophoresis and Nanodrop 2000 spectrophotometer (Nanodrop, USA). cDNA library construction was performed at Shanghai Majorbio Bio-pharm Biotechnology Co., Ltd. (Shanghai, China). Briefly, Oligo-dT beads were used to isolate poly(A_+_) mRNA from a total RNA pool consisting of equal quantities of total RNA from three sample types: untreated susceptible barnyardgrass, untreated resistant barnyardgrass and herbicide-treated resistant barnyardgrass. Then, mRNA was converted into cDNA using the SMART cDNA synthesis kit (Clontech, USA) with a modified 3′ primer containing a *Bsg*I site (5′-ATTCTAGAGGCCGAGGCGTGCAG d(T)18 VN-3′). The newly synthesized cDNA were treated with *Bsg*I enzyme to remove the poly (A) tails, fragmented, blunt ended and ligated with the customized 454 adaptors (A and B) to form the single-stranded cDNA library for emulsion PCR amplification and Roche GS-FLX 454 sequencing.

### 454 Sequencing, Assembly and Annotation

The cDNA pool representing untreated susceptible barnyardgrass, untreated resistant barnyardgrass and herbicide-treated resistant barnyardgrass was used for high throughput sequencing using the 454 GS-FLX platform (Roche, USA). A half-plate run was performed at the Shanghai Majorbio Bio-pharm Biotechnology Co., Ltd. (Shanghai, China) following the manufacturer’s protocol. GS *de novo* assembler (Newbler v2.3, Roche) was used for 454 sequencing assembly. For similarity searches, all assembled contigs and singletons were compared with the proteins in the NCBI non-redundant (nr) protein database using BLASTx [20]. The BLAST E-value threshold was set at 1×10^−5^, with a minimum alignment length of 60 bp. To determine gene functions of the sequences, these results were imported to Blast2GO, a software that retrieves gene ontology (GO) terms [21]. GO terms were determined with respect to Cellular Component, Biological Process and Molecular Function. Kyoto Encyclopaedia of Genes and Genomes (KEGG) pathways were assigned to the assembled sequences and Enzyme Commission (EC) numbers were used to automatically color and retrieve KEGG pathway maps [22]. The assembled sequences were mapped to the KEGG biochemical pathways according to the enzyme distribution in the pathway database. InterPro annotations were performed using InterProScan (http://www.ebi.ac.uk/Tools/InterProScan/).

### Illumina Sequencing and Digital Expression Analysis

Oligo-dT beads were used to yield poly(A_+_) mRNA from a total RNA pool consisting of equal quantities of total RNA from four sample types: untreated susceptible barnyardgrass, herbicide-treated susceptible barnyardgrass, untreated resistant barnyardgrass and herbicide-treated resistant barnyardgrass. Then, mRNA bound to the beads was converted into first-strand cDNA using oligo-dT and second-strand cDNA using random primers. Paired-ended (PE) library with approximate 200 bp fragment was synthesized using the Genomic DNA Sample Prep Kit (Illumina, USA) according to the manufacuter’s instructions. PE library products were sequenced on the Illumina Genome Analyzer II platform equipped with a paired-end module according to the manufacturer’s instruction. Then the PE reads were assembled using ABySS assembler tool [23]. The sequences were aligned to the assembled contigs and singletons using the bowtie software [24]. The expressions of each reads between sample pairs (untreated susceptible barnyardgrass VS herbicide-treated susceptible barnyardgrass, untreated resistant barnyardgrass VS herbicide-treated resistant barnyardgrass) were calculated using the numbers of reads with a specific match. Among the four samples, a minimum of a two-fold difference in log2 expression were considered as expression differences. Genes with different expression were identified using R package DEGseq with MARS (MA-plot-based method with random sampling Model) [25].

### Data Availability

All the reads generated in 454 pyrosequencing and Illumina sequencing were deposited in the NCBI Sequence Read Archive (SRA) under the accession number SRX160526.

### Quantitative Real-time PCR Analysis

Quantitative real-time PCR analysis of two candidate genes, *ALS* and *ACCase*, was performed using gene specific primers. *ALS* primers were 5′-GCCTTCCAGGAGACGCCAATCG-3′ and 5′-GGTCATCTCCACAAAGCGGCACA-3′. *ACCase* primers were 5′-GCTGCGTGGAGGAGCTTGGG-3′ and 5′-CACCTTTAGCTGCCATTCTGAGGGA-3′. The *β-actin* gene was used as an internal control. The primers used to amplify *β-actin* were 5′-CACACTGGTGTTATGGTAGG-3′ and 5′-AGAAGGTGTGATGCCAAAT-3′. Real-time PCR was performed using SYBR® *Premix Ex Taq*™ kit (TaKaRa, Japan) on a ABI-7500 Fast Real-Time PCR System (ABI, USA). About 2µl cDNA from each of the four samples (untreated and herbicide-treated susceptible barnyardgrass, untreated and herbicide-treated resistant barnyardgrass) was mixed with 0.2 µM primers, 1×ROX Reference Dye II and SYBR® *Premix Ex Taq*™ (including TaKaRa Ex Taq HS, dNTP mixture, Mg^2+^ and SYBR Green I) in a final volume of 20 µl. The amplification program was as follows: 30 s at 95°C for denaturation followed by 40 cycles of 3 s at 95°C and 30 s at 60°C. Each experiment had three independent repetitions. Values were presented as mean ± SD of three independent RT-PCR analysis. Error bars were calculated based on values from three replicates. Relative expression of the target genes to the *β-actin* control was calculated using the 2^−△△CT^ method [26].

## Results and Discussion

### 454 Sequencing and *de novo* Assembly

**Figure 2:**
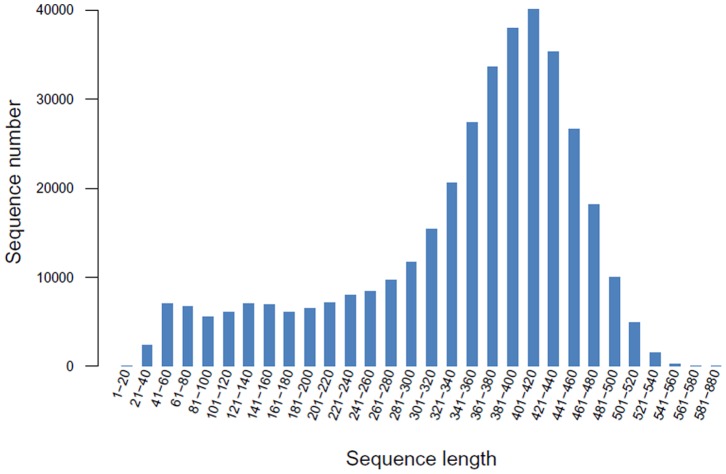
Distribution of the raw read sequence lengths from the barnyardgrass 454 transcriptome.

We performed 454 pyrosequencing of the barnyardgrass (*E. crus-galli*) cDNA to identify target genes for functional genome studies. Our choice to sequence the transcriptome was based on the difficulty in sequencing the polyploid barnyardgrass genome by whole genome sequencing. A pooled cDNA library from three samples (untreated susceptible biotype, untreated resistant biotype and herbicide-treated resistant biotype) was run on a half-plate using the 454 GS-FLX Titanium Chemistry to obtain sequences of low- and high- abundance transcripts. This biotype-treatment combo could produce the transcripts related to herbicide resistance, herbicide-treated susceptible biotype was therefore absent in this 454 pyrosequencing,. This generated 371,281 raw reads with an average length of 341.8 bp and a total length of 126.89 Mb (Table 1). Sequence length distribution of raw reads is shown in Fig. 2.

**Figure 3:**
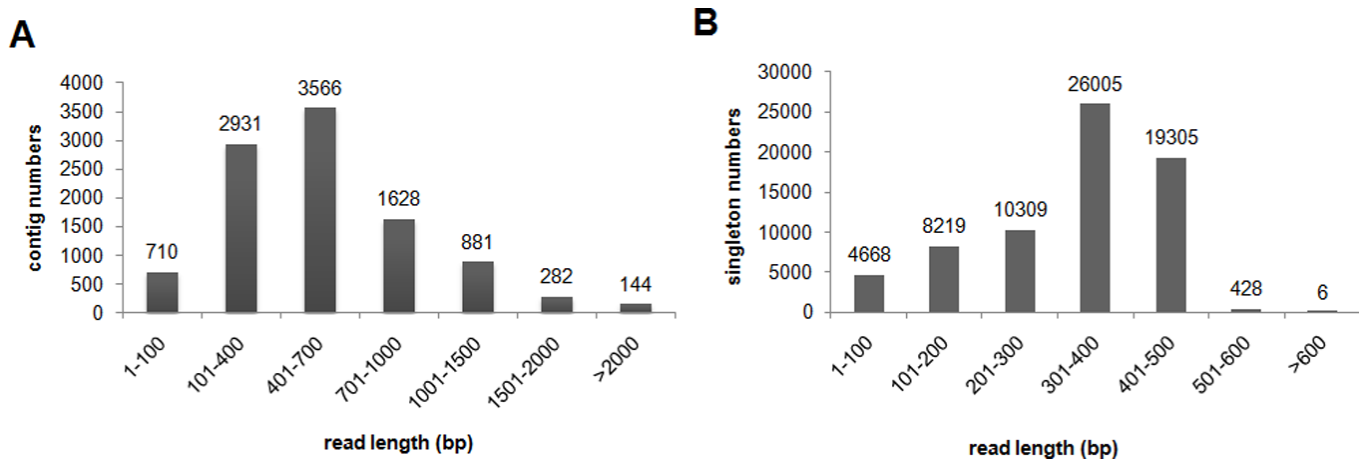
Sequence length distribution of assembled contigs (A) and singletons (B) from the barnyardgrass 454 transcriptome.

### Sequence Annotation

In order to identify new *E. crus-galli* specific sequences, all 79,082 assembled sequences (10,142 contigs and 68,940 singletons) were used as queries to search the NCBI nr protein database using BLASTx followed by analysis using Blast2GO. In total 45,438 assembled sequences including 7,407 (73.03%) contigs and 38,031 (55.17%) singletons were annotated. The remaining 44.83% singletons and 26.97% contigs were not annotated presumably due to their short average lengths. Average length of the annotated contigs was 690 bp, while that of non-annotated contigs was only 260 bp. The average length of annotated singletons was 360 bp, while that of non-annotated singletons was 272 bp.

In these annotations, only 24 sequences were hits to proteins from the *Echinochloa* species while 23,657 (52.06%) were hits to proteins from *Sorghum bicolor*; 9,584 (21.09%) to *Zea mays*; 7,718 (16.99%) to *Oryza sativa* and 1,764 (3.88%) to *Hordeum vulgare*. Such low numbers of proteins from barnyardgrass is most likely due to the absence of a well annotated reference genome for the species. Almost half of the sequences (42.54%) assembled in this study could not be annotated by searching the NCBI nr protein database. Absence of sufficient sequences from species phylogenetically close to barnyardgrass in the public databases could have also contributed to the high percentage of non-annotated sequences. These non-annotated sequences could be resourceful for pursuing taxon-guided searches of specific genes of interest [15].

**Figure 4:**
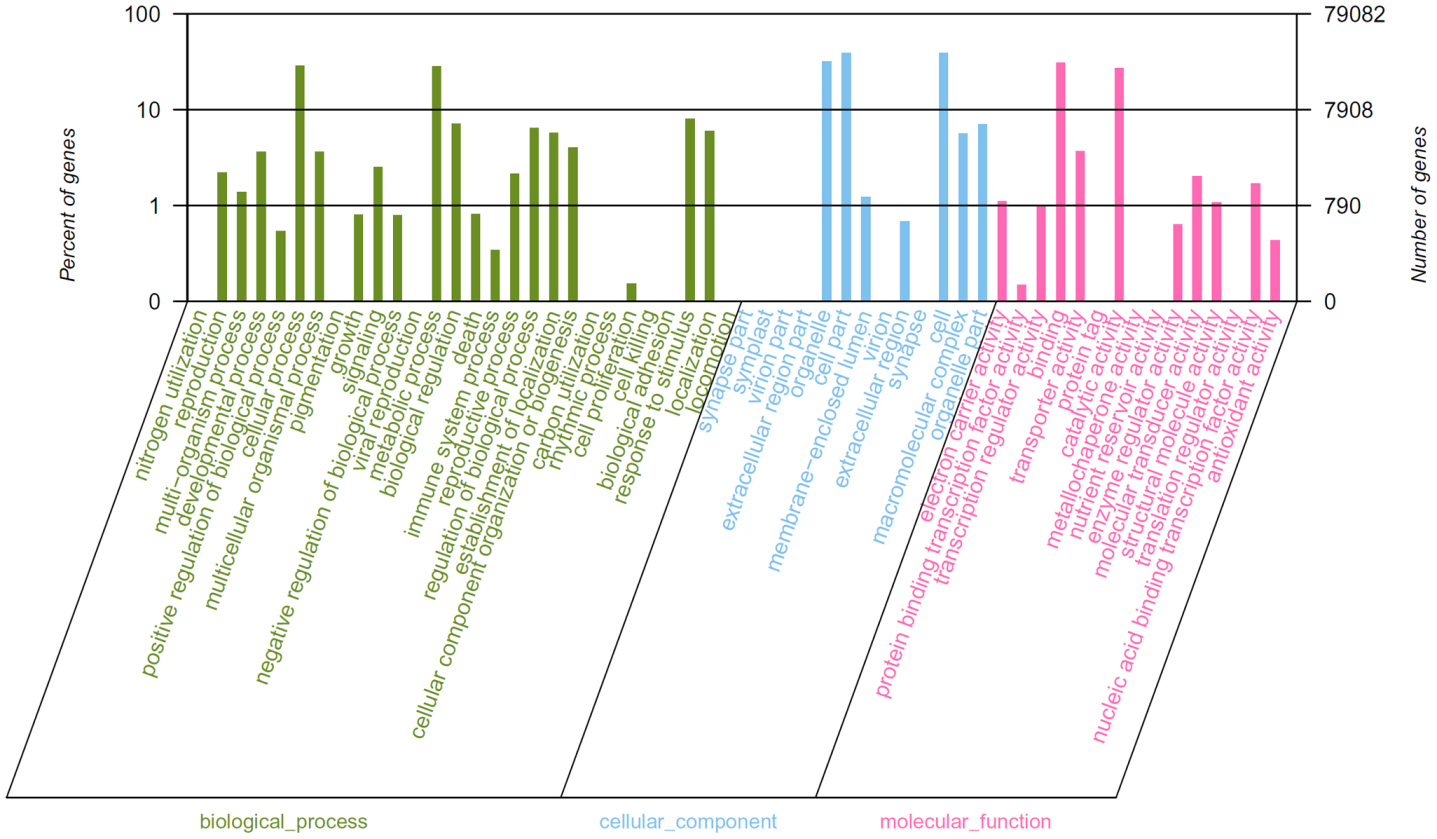
Functional annotation of assembled sequences based on gene ontology (GO) terms. GO analysis was performed at level 2 for the three main categories (biological process, cellular component and molecular function).

GO terms were assigned to the annotated EST sequences using Blast2GO. One or more GO terms were assigned to 40,399 (51.09%) sequences with 244,653 GO assignments in total for the biological process, cellular component and molecular function categories. These three groups were not mutually exclusive because many contigs were assigned to more than one type of GO term. The largest proportion was represented by the cellular process (GO: 0009987; 25.16%) and metabolic process (GO: 0008152; 25.05%) under biological process, cell (GO: 0005623; 31.40%), cell part (GO: 0044464; 31.40%) and organelle (GO:0043226; 25.40%) under cellular component, and binding (GO: 0005488; 44.36%) and catalytic activity (GO: 0003824; 38.66%) under molecular function (Fig. 4). Representation of 56 GO terms in the transcriptome provided an indication of the diversity of genes expressed in *E. crus-galli*. Similar results were reported for the biological process category in *Conyza canadensis,* while the predominant subgroups in molecular function (enzyme activity and transferase activity) and cellular component (intracellular components and cytoplasmic components) are different from our data [16]. To our interests were the GO terms related to herbicidal mechanisms: response to auxin stimulus (152), photosystem II (114), cellulose biosynthetic process (106), lipid catabolic process synthesis (95), acetyl-CoA biosynthetic process (65), carotenoid biosynthetic process (37) and acetolactate synthase activity (6). Quinclorac used in our study is an inhibitor of synthetic auxin (acting like indolylacetic acid) and also an inhibitor of cell wall (cellulose) biosynthesis [4]. About 51 sequences under auxin response factor and 28 sequences under cellulose synthase were obtained. These sequences could be further investigated to determine their specific function in the evolution of herbicide resistance in *E. crus-galli*.

### Functional Classification by KEGG

In addition, KEGG pathways were assigned to all 79,082 assembled sequences by comparing to the KEGG database using BLASTx with an E-value cutoff of <1×10^−5^. A total of 6,092 contigs and singletons with 2,515 enzyme commission (EC) numbers were assigned to 151 predicted KEGG metabolic pathways and the numbers of barnyardgrass sequences in the different pathways ranged from 1 to 4,424. This analysis showed that 4,424 sequences were present in the major metabolic pathways and 1,865 in the biosynthesis of secondary metabolites. The sequences represented in the metabolic pathways of major bio-molecules included purine, starch and sucrose, terpenoids and steroids, methane, pyrimidine, phenylalanine, amino sugar and nucleotide sugar, glycerophospholipid, glycerolipid, etc. About 894 sequences with 109 EC numbers were mapped to the biosynthesis of plant hormones pathway. In the metabolism pathway, we found seven sequences assigned to acetolactate synthase (ALS, EC 2.2.1.6), which is the target for herbicides, penoxsulam and bispyribac-sodium. When these sequences were compared in the NCBI nr database using BLASTx, only two (contig05544 and G81ESPA01CWAJO) sequences were found to be highly similar (99% and 99%) to ALS genes from *Echinochloa phyllopogon* [28], while the remaining five (contig03089, contig06883, contig08547, G81ESPA01BLGZC and G81ESPA01ECFXF) were annotated to hypothetical proteins of unknown function.

### Illumina Sequencing and Digital Expression Analysis

**Figure 5:**
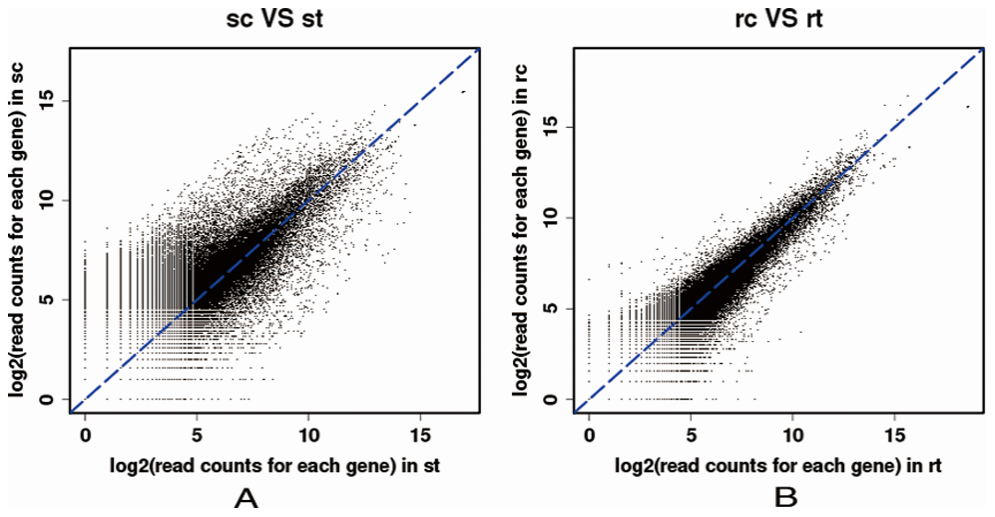
Scatter plot analysis of two sample pairs (sc VS st, rc VS rt) from barnyardgrass. sc: untreated susceptible biotype; st: herbicide-treated susceptible biotype; rc: untreated resistant biotype; rt: herbicide-treated resistant biotype. Log2 transformed read counts of the mapped contigs and singletons were used for comparison.

To identify large scale differences in gene expression of susceptible and resistant barnyardgrass, quantitative transcriptome analysis using Illumina Genome Analyzer II was performed. It produced quantitative expression scores, which should be accurate and highly consistent with other quantitative methods such as quantitative RT-PCR [29]. A mixed cDNA pool from four samples (untreated and herbicide-treated susceptible barnyardgrass, untreated and herbicide-treated resistant barnyardgrass) was sequenced using Illumina. This yielded 2.8 GB data with 14,769,229, 14,610,811, 12,341,146 and 15,099,517 assembled paired-ended reads respectively. These transcripts were mapped to the assembled sequences from 454 pyrosequencing and those that were mapped successfully were further used to determine and compare the expression profiles of the transcripts in the four samples. Digital expression analysis identified a total of 78,124 sequences from 454 pyrosequencing including 9,737 contigs and 68,387 singletons showing significant abundance differences between sample pairs (untreated susceptible barnyardgrass VS herbicide-treated susceptible barnyardgrass, untreated resistant barnyardgrass VS herbicide-treated resistant barnyardgrass) (Fig. 5). The expression of many non-annotated genes with unknown function was revealed in this digital gene expression analysis. Thus this transcriptome could be a potentially rich source of genetic material for the systematic discovery of genes involved in the mechanisms of herbicide resistance [17].

In the digital gene expression analysis, we found that asparagines synthetase 2 had the highest expression in the herbicide-treated resistant barnyardgrass. The top 10 annotated sequences with highest expression level in the barnyardgrass transcriptome are listed in Table 2. Except sucrose synthase, the other nine genes had higher expression in herbicide-treated resistant barnyardgrass than in herbicide-treated susceptible barnyardgrass biotype. Metallothionein-like protein, elongation factor, acetyl-coenzyme A carboxylase, light-induced protein 1 and pyruvate orthophosphate dikinase had higher expression levels in the herbicide-treated resistant barnyardgrass than the untreated resistant barnyardgrass biotype. In addition, the expression levels of these genes were lower in the herbicide-treated susceptible barnyardgrass and lowest in the untreated susceptible barnyardgrass biotype. Functional analysis of these genes will be conducted in future to determine the relationship between these genes and herbicide resistance.

**Table 2:** The top 15 genes with highest expression levels in the transcriptome data.

No.	Sequence ID	Annotation	Digital expression			
			SC	ST	RC	RT
1	contig04556	asparagines synthetase 2	8,433	6,834	108,302	50,444
2	contig04555	asparagines synthetase 2	5,984	4,222	74,988	39,573
3	contig06106	glycine-rich RNA-binding protein 2	7,229	4,267	20,464	18,167
4	contig07670	metallothionein-like protein	9,599	498	10,924	16,650
5	contig03094	light-induced protein 1-like	15,289	4,400	14,736	14,398
6	contig04500	sucrose synthase 1	7,879	13,895	8,938	12,881
7	contig00684	pyruvate orthophosphate dikinase	22,739	7,861	5,464	12,814
8	contig00581	elongation factor	15,557	6,714	11,676	12,365
9	contig04470	acetyl-coenzyme A carboxylase	16,114	2,472	12,989	12,088
10	contig05353	light-induced protein 1-like	11,779	3,203	13,083	11,876

### Comparison of the Sequences Involved in Herbicide Resistance

The candidate genes involved in herbicide resistance were obtained by comparing our transcriptome (454) with the trascriptomes of waterhemp [15], horseweed [16] and grain amaranth [17]. The comparison of eleven herbicide target gene families and four non-target gene families is presented in Table 3. More glutamine synthetase (16) and phytoene desaturase (7) transcripts from the herbicide target gene families were identified in barnyardgrass than in the other three species. Notable absence in the barnyardgrass transcriptome were 4-hydroxyphenylpyruvate dioxygenase, protophenylpyruvate dioxygenase and D1 protein. The reason of such absence could be due to similar expression patterns between the susceptible and resistant biotypes. From our analysis we could conclude that 215 assembled sequences could be involved in the evolution of herbicide resistance. Since there is no reference genome to assemble these short reads into scaffolds and these 215 sequences could be redundant, the actual number of genes could be less than 215. In future work, we will focus on the functional analysis of these candidate genes.

**Table 3:** Comparison of the herbicide target gene families and non-target gene families from transcriptome of barnyardgrass, waterhemp, horseweed and grain amaranth.

	Barnyardgrass	Waterhemp	Horseweed	Grain amaranth
**Herbicide target gene family**				
tubulin	25	33	29	34
glutamine synthetase	16	7	9	11
acetyl-CoA carboxylase	8	8	6	13
phytoene desaturase	7	1	5	2
1-deoxy-D-xylulose-5-phosphate synthase	3	1	6	3
5-enolpyruvylshikimate-3-phosphate synthase	2	3	2	1
acetolactate synthase	2	2	6	5
dihydropteroate synthase	1	2	1	1
4-hydroxyphenylpyruvate dioxygenase	0	2	1	3
protoporphyrinogen oxidase	0	8	2	5
D1 protein (plastidic gene)	0	2	4	2
**Non-target gene family**				
cytochrome P450 monooxygenase	36	191	125	–
glycosyltransferase	31	84	76	–
ABC transporter genes	64	192	151	–
glutathione S-transferase	19	22	7	–

**Figure 6:**
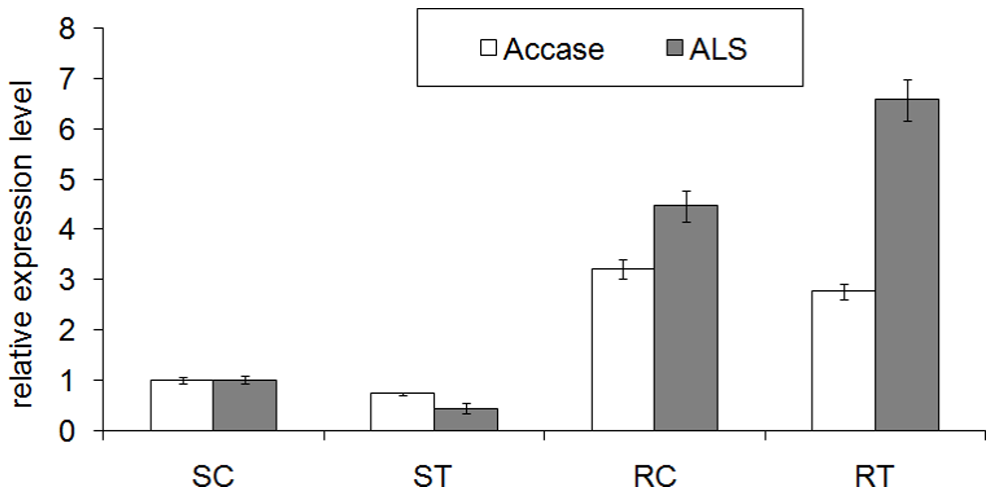
Real-time PCR analysis of *ALS* and *ACCase* genes in the seedlings of barnyardgrass. ALS: Acetolactate synthase; ACCase: Acetyl-CoA carboxylase; SC: untreated susceptible biotype; ST: herbicide-treated susceptible biotype; RC: untreated resistant biotype; RT: herbicide-treated resistant biotype. The *β-actin* gene was used as an internal control. Values represent mean ± SD. Error bars were calculated based on values from three replicates.

Microtubules are polymers of α-tubulin and β-tubulin dimers and play an important role in many essential cellular processes including mitosis, cytokinesis and vesicular transport [3]. The digital expression of 24 tubulin genes including α-tubulin and β-tubulin was much higher in the herbicide-treated resistant biotype (RT) than the herbicide-treated susceptible biotype (ST) of *E. crus-galli*, except one α-tubulin gene (G81ESPA01BDRNJ) and three β-tubulin genes (G81ESPA01CF9IL, G81ESPA01BZVDB and G81ESPA01BQRNR) with lower expression (Table 4). After treatment with herbicides, the digital expression levels of tubulin genes in the susceptible biotype (ST) declined when compared with untreated susceptible biotype (SC). However, the digital expressions of tubulin genes in the resistant biotype remained the same or declined below the susceptible biotype. The microtubule growth was more disrupted in the susceptible biotype than in the resistant biotype after the herbicide treatment. Tubulin genes play an important role in herbicide induced growth inhibition. For instance, in goosegrass (*Eleusine indica*), nucleotide differences were observed in the three α-tubulin genes between resistant (R) and susceptible (S) biotypes, although missense mutations were not present in any of the four β-tubulin genes in the two R-biotype lines [30], [31]. This indicated the possible association between these missense mutations in the α-tubulin genes and herbicide resistance in goosegrass. Similar results were found between the resistant and sensitive biotypes of green foxtail (*Setaria viridis* L. Beauv.) [32]. Therefore, the isolation and functional analysis of the full-length α-tubulin and β-tubulin genes from barnyardgrass is necessary to understand the mechanisms of herbicide resistance. Acetyl-CoA carboxylase (ACCase, EC 6.4.1.2) is a key enzyme in lipid biosynthesis that catalyzes the formation of malonyl-CoA from acetyl-CoA [3]. Similar to tubulin genes, the digital expression of eight ACCase genes also showed higher expression in the resistant biotype than in the susceptible biotype after herbicide treatment (Table 5). Two ACCase belonging to *E. crus-galli* biotype Geqiushan-S (HQ395758) and Geqiushan-R (HQ395759) were found in NCBI. These genes shared 96% identity with contig04470 and no significant similarity with contig04540. Further studies on the ACCase genes in barnyardgrass are needed to compare the differences among these genes, and determine their role in herbicide resistance. Penoxsulam and bispyribac-sodium herbicides, which were used in our study, were ALS-inhibiting herbicides [33]. In the barnyardgrass transcriptome and digital expression analysis, higher levels of ALS sequences (contig05544 and G81ESPA01CWAJO) were present in the resistant biotype than in the susceptible biotype after treatment with herbicides (Table 6). Based on real-time PCR experiments, the relative expression of *ACCase* and *ALS* genes were analyzed in each untreated and herbicide-treated biotype. Compared to herbicide treated susceptible plants, *ALS* and *ACCase* were highly expressed in herbicide treated resistant plants. The expressions of *ALS* and *ACCase* genes decreased when the susceptible biotype was treated with herbicides. However, the expression of *ACCase* declined slightly and *ALS* increased slightly in the herbicide-treated resistant biotype (Fig. 6). This trend was consistent with the expression profiles of ALS and ACCase sequences in the transcriptome. These genes are therefore regarded as good preliminary targets for further functional studies of herbicide resistance.

**Table 4:** Sequence ID, annotation, hit size and digital expression profile of tubulin genes in barnyardgrass (*E. crus-galli*).

No.	Sequence ID	Annotation	Hitsize (bp)	Digitalexpression			
				SC	ST	RC	RT
1	contig02007	α-tubulin	447	4,561	429	5,173	5,029
2	contig02008	α-tubulin	935	10,817	1,185	8,054	7,396
3	contig02010	α-tubulin	935	9,359	1,043	7,354	7,069
4	contig02031	α-tubulin	911	6,782	890	5,483	6,575
5	contig02033	α-tubulin	309	1,276	190	1,147	1,266
6	contig02034	α-tubulin	330	1,252	221	1,253	1,502
7	contig04855	α-tubulin	1,461	4,939	1,449	2,076	3,832
8	contig04932	α-tubulin	1,364	3,400	1,509	5,048	3,828
9	G81ESPA01D3IDY	α-tubulin	439	1,203	209	921	1,141
10	G81ESPA01BB9ZQ	α-tubulin	364	1,304	167	839	814
11	G81ESPA01AXQ2P	α-tubulin	129	1,489	232	1,288	1,270
12	G81ESPA01BU5X0	α-tubulin	305	1,084	191	888	1,097
13	G81ESPA01DWSAD	α-tubulin	387	1,206	224	517	560
14	G81ESPA01D0ZJ4	α-tubulin	461	1,609	228	1,042	1,034
15	G81ESPA01D6KVH	α-tubulin	366	258	217	358	275
16	G81ESPA01AG82W	α-tubulin	181	1,350	220	1,181	1,138
17	G81ESPA01BDRNJ	α-tubulin	349	1	2	6	3
18	contig00720	β-tubulin	415	508	185	849	491
19	contig00721	β-tubulin	603	2,198	270	1,597	1,517
20	contig00723	β-tubulin	681	1,514	123	837	1,126
21	contig00724	β-tubulin	939	2,136	591	2,308	1,695
22	contig00725	β- tubulin	789	1,608	603	1,347	1,127
23	G81ESPA01CF9IL	β- tubulin	291	112	18	42	53
24	G81ESPA01BZVDB	β- tubulin	283	99	14	31	42
25	G81ESPA01BQRNR	β- tubulin	161	67	12	23	27

SC: untreated susceptible biotype; ST: herbicide-treated susceptible biotype; RC: untreated resistant biotype; RT: herbicide-treated resistant biotype.

**Table 5:** Sequence ID, annotation, hit size and digital expression profile of acetyl-CoA carboxylase.

No.	Sequence ID	Hit size(bp)	Digital expression			
			SC	ST	RC	RT
1	contig04470	6,708	16,114	2,472	12,989	12,088
2	contig04540	2,225	2,903	1,495	1,379	1,515
3	G81ESPA01C7K8F	295	74	28	59	88
4	G81ESPA01EALO0	355	103	37	78	113
5	G81ESPA01DANWF	346	410	113	283	276
6	G81ESPA01D05V2	221	135	22	145	121
7	G81ESPA01CVZK6	301	350	50	263	242
8	G81ESPA01CW4DK	438	479	78	513	478

**Table 6:** Sequence ID, annotation, hit size and digital expression profile of acetolactate synthase.

No.	SequenceID	Annotation	Hitsize (bp)	Digitalexpression			
				SC	ST	RC	RT
1	contig05544	acetolactate synthase	991	482	143	557	385
2	G81ESPA01CWAJ0	acetolactate synthase	409	233	28	271	235

### Comparison of the Genes in *Echinochloa*

About 26 assembled sequences had best hits to *Echinochloa* genes in the NCBI nr database and included *ALS* [34], *ACCase* [28], NADP-dependent malic enzyme (*NADP-me*), pyruvate orthophosphate dikinase (*PPDK*) [35], *Rps3*, maturase K (*matK*) [36], [37], phosphoenolpyruvate carboxylase (*PEPC*) [38] and ribulose-1,5-bisphosphate carboxylase/oxygenase large subunit (*rbcL*) [39]. These seven genes were obtained from different varieties of *Echinochloa* including *E. phyllopogon*, *E. frumentacea*, *E. colona*, *E. esculenta*, *E. muricata* and *E. oryzicola*, in addition to *E. crus-galli* (Table 7). According to our barnyardgrass transcriptome data, C_4_ photosynthesis genes with complete coding sequence (CDS) including ribulose-1,5-bisphosphate carboxylase/oxygenase small subunit (*rbcS*) (KC478601), *rbcL* (KC478602), *NADP-me* (KC478603) and malate dehydrogenase (*MDH*) (JX683695) were identified from *E. crus-galli* using PCR and RACE technology. These genes in general will be benefit future genomic and phylogenetic studies of grasses. Functional analysis of these genes is necessary to understand the evolution of C_4_ photosynthesis during angiosperm diversification and phylogenetic patterns in grasses [38], [40].

**Table 7:** A list of *Echinochloa* genes with homology to barnyardgrass sequences.

*Echinochloa*	Gene	NCBI accession number
***E. crus-galli***	ALS	JQ319776, JX415268∼JX415271[^34^]
	ACCase	AY763585, AH014492, AJ966453, AJ966423, AY762963, HQ395758, HQ395759
	NADP-me	FJ603315, FJ644948
	PPDK	DQ083757
	Rps3	FJ766298, FJ766300
	matK	HM850573, HQ593274, HQ599953, HQ599954, HQ599962
	PEPC	AY251482, AY995212
	rbcL	HQ590071
***E. phyllopogon***	ALS	AB636580, AB636581[^28^]
	ACCase	AB636582∼AB636588[^28^]
***E. frumentacea***	PPDK	AY792619, AB289641[^35^]
***E. colona***	matK	HM850572[^36^]
***E. esculenta***	matK	AF164422[^37^]
	PEPC	AM690215[^38^]
	rcbL	EF125128[^39^]
***E. muricata***	matK	HQ593275, HQ596677
	rcbL	HQ590072
***E. oryzicola***	Rps3	FJ766299

### Conclusions

The susceptible and resistant biotypes of *E. crus-galli*, with and without treatment with three herbicides, were used to generate the first large-scale transcriptome sequencing data using the 454 GS-FLX platform. *De novo* assembly and functional annotation of the assembled contigs and singletons were performed using Newbler. Interestingly, eight herbicide target-site gene groups and four non-target-site gene groups were identified in the resistant biotype of *E. crus-galli* that would enable isolation of the full-length genes, which could be involved in herbicide resistance. These genes could also be used for further functional genomics research. Compared to the previously *Echinochloa* reported genes, four additional genes including *RbcS*, *RbcL*, *NADP-me* and *MDH* with complete CDS were identified using PCR and RACE technology. This study provides a valuable genetic resource for grasses in general and should be useful for weed control which should result in an improvement in agriculture.
